# Physical Layer Key Generation in 5G and Beyond Wireless Communications: Challenges and Opportunities

**DOI:** 10.3390/e21050497

**Published:** 2019-05-15

**Authors:** Guyue Li, Chen Sun, Junqing Zhang, Eduard Jorswieck, Bin Xiao, Aiqun Hu

**Affiliations:** 1 School of Cyber Science and Engineering, Southeast University, Nanjing 210096, China; 2 The National Mobile Communications Research Laboratory, School of Information Science and Engineering, Southeast University, Nanjing 210096, China; 3 Department of Electrical Engineering and Electronics, University of Liverpool, Liverpool L69 3GJ, UK; 4 Communications Laboratory, Technische Universität Dresden, 01062 Dresden, Germany; 5 Department of Computing, The Hong Kong Polytechnic University, Hung Hom, Kowloon, Hong Kong

**Keywords:** physical layer security, secret key generation, in-band full-duplex, massive MIMO, mmWave communications, IoT prototypes

## Abstract

The fifth generation (5G) and beyond wireless communications will transform many exciting applications and trigger massive data connections with private, confidential, and sensitive information. The security of wireless communications is conventionally established by cryptographic schemes and protocols in which the secret key distribution is one of the essential primitives. However, traditional cryptography-based key distribution protocols might be challenged in the 5G and beyond communications because of special features such as device-to-device and heterogeneous communications, and ultra-low latency requirements. Channel reciprocity-based key generation (CRKG) is an emerging physical layer-based technique to establish secret keys between devices. This article reviews CRKG when the 5G and beyond networks employ three candidate technologies: duplex modes, massive multiple-input multiple-output (MIMO) and mmWave communications. We identify the opportunities and challenges for CRKG and provide corresponding solutions. To further demonstrate the feasibility of CRKG in practical communication systems, we overview existing prototypes with different IoT protocols and examine their performance in real-world environments. This article shows the feasibility and promising performances of CRKG with the potential to be commercialized.

## 1. Introduction

The 5G and beyond wireless communications will support massive transmission of important and private information, including personal data, financial information, military secrets and mission-critical industrial control messages [1,2]. Communication security is one of the top priorities in future wireless network design [3]. To safeguard the data transmission, public key cryptography techniques and associated protocols are widely applied to distribute secret keys among users and devices. However, these solutions are facing new challenges in 5G and beyond wireless communications: they may be cracked by the emerging quantum computers in the future [4]; they require a public key infrastructure which may not be available in many 5G applications such as device-to-device communications [5]; they introduce latency which may not meet the ultra low latency requirements [6]; and they face difficulties of key distribution and management among heterogeneous Internet of Things (IoT) devices [7].

### 1.1. Fundamentals of Physical Layer Key Generation

Physical layer key generation has recently emerged as a new paradigm that provides a lightweight and information-theoretic secure key sharing solution [8,9,10,11,12,13]. It is built on the basis of channel reciprocity, which means that the channel responses of the forward and backward communication links are the same (or very similar) [14]. Besides, the dynamic, time-varying or randomly changing and complex wireless communication environment makes the channel responses change over time and hard to predict [15]. Inspired by this, legitimate users can share a pair of common randomness by estimating the channel between them. Since physical layer key generation does not require assistance from a third party, it can be deployed in parallel in massive connections.

**Figure 1:**
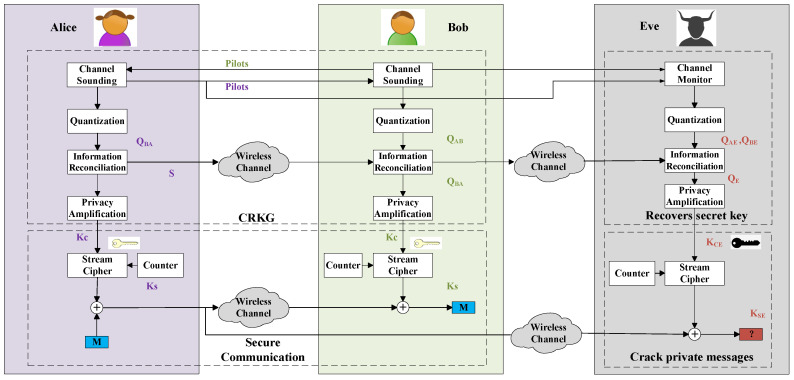
An example of secure transmission exploiting the key produced by CRKG.

Physical layer key generation establishes secret keys between two legitimate users, named Alice and Bob, from the common randomness of their wireless channel. As shown in Figure 1, the key generation procedure involves four stages: channel probing, quantization, information reconciliation, and privacy amplification. Alice and Bob probe the wireless channel alternately to obtain correlated channel measurements, which is the key step to harvest channel randomness and is the focus of this article. Key generation relies on the reciprocal channel features in the time, frequency, and spatial domains. We therefore term this technique as channel reciprocity-based key generation (CRKG). Quantization converts these analog channel measurements into digital binaries, i.e., $Q_{BA}$ at Alice and $Q_{AB}$ at Bob. It is worth noting that, when the two probes are within the same coherence time and/or coherence bandwidth, the observed channel information are self-correlated. Therefore, adjacent channel features are likely to have the same quantization value, which leads to long 0 s or 1 s and reduces key randomness. To address this problem, a preprocessing step is usually added between channel sounding and quantization. Various preprocessing approaches have been proposed to reduce the correlation and also to enhance the similarity of the channel features [16]. Next, information reconciliation enables Alice and Bob to agree on the same key through error detection protocols or error correction codes [17]. Finally, privacy amplification eliminates any potential information leakage to eavesdroppers. More details of the procedure can be found in [10]. Therefore, it is necessary to develop infrastructure-less, fast and lightweight secret key generation algorithms.

Figure 1 also illustrates an example of safeguarding data transmission exploiting the key produced by CRKG. Alice and Bob share a pair of symmetric channel key $K_{c}$ through CRKG described above. Next, these keys are fed to a stream cipher as the seed to produce session keys to encrypt the plaintext. A pair of $K_{c}$ may be used multiple times, but the session keys are different due to the increase of the counter number. The seed can be updated when another pair of channel keys are generated. The secure transmission scheme based on CRKG is lightweight as it does not introduce additional complexity. Therefore, it is very quite suitable for IoT applications.

Meanwhile, a passive eavesdropper (Eve) can also observe the whole procedure of CRKG. Based on the wireless decorrelation property of wireless channels, when an eavesdropper is more than half-wavelength away from the legitimate users, it will experience statistically independent channel fading, and thus cannot infer the generated key. In this case, Eve obtains the quantization of channel information from Alice to Eve and from Bob to Eve as $Q_{AE}$ and $Q_{BE}$. Similarly, she can generate secret key $K_{CE}$. The secret key $K_{CE}$ is not consistent with $K_{c}$, and she cannot crack private messages. A detailed discussion of security issues of CRKG under practical passive and active attacks can be found in [9].

### 1.2. Our Contributions

Many excellent tutorials [8,9] and surveys [6,10] have been published in physical layer key generation, which provide comprehensive overview and insightful comments to understand the fundamental principles, procedures, applications and security issues in this field. The focused issues and the main contents of some published surveys and tutorials are highlighted in Table 1.

**Figure 2:**
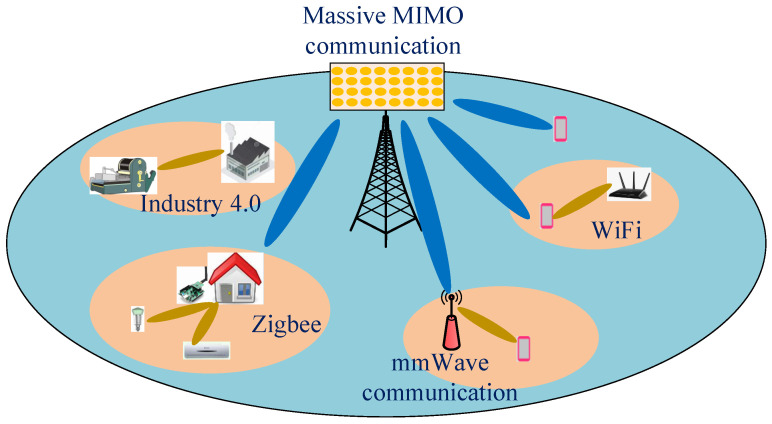
New air interference technologies and potential applications of CRKG in 5G and beyond.

In contrast to existing surveys and and tutorials, our work aims to clarify the feasibility and scalability of CRKG to support new air interface technologies, e.g., full duplex, massive MIMO, mmWave, and heterogeneous IoT devices in the 5G and beyond. These new air interface technologies make it more convenient to extract security elements from wireless resources. Thereby, this paper brings CRKG closer to practical dissemination and products applications, as illustrated in Figure 2. The main contributions of the article are summarized as follows:

- **CRKG with Various Duplex Modes**: We are the first to investigate the fundamental performance and challenges of key generation with various relevant duplex modes, including Time Division Duplex (TDD), Frequency Division Duplex (FDD) and In-band Full-Duplex (IBFD). In TDD systems, the key generation rate is limited by the velocity of users. Under FDD operation, due to the summation of different propagation paths, the channel responses are generally not similar. To cope with this problem, we provide a solution by separating different propagation paths. Although IBFD enables wireless users to transmit and receive simultaneously over the same frequency band, it brings new challenges due to the large self-interference. Finally, we compare the secret key rates of these three duplex modes under the influence of the velocity, frequency interval and residual self-interference.
- **CRKG With Massive MIMO and mmWave**: We provide a new study on how to increase the secret key rate of CRKG by leveraging the advantages of massive MIMO and mmWave. The impact of number of antennas on secret key rates is analyzed and demonstrated through simulations. In addition, we point out some essential problems, including the channel state information (CSI) acquisition and pilot contamination attacks in massive MIMO and severe path loss and penetration loss in mmWave communications. We also provide potential solutions by employing some specific channel characteristics, e.g., sparsity in massive MIMO and non-stationary in mmWave.
- **CRKG Prototypes in the IoT**: We review some existing practical explorations and verify the scalability of CRKG among heterogeneous IoT devices. IoT is expected to play an essential role within future 5G and beyond systems. We select four typical wireless communication technologies that are common in IoT, including ZigBee, ultra-wideband and WiFi for short-range applications and LoRa for long-range applications. Furthermore, we discuss several open research challenges and provide some new research directions of CRKG in the future IoT networks.

The rest of the article is organized as follows. The performance and challenges of key generation with three typical duplex modes are discussed in Section 2. We study the CRKG by leveraging the advantages of massive MIMO and mmWave in Section 3, and bring CRKG into securing IoT communications in Section 4. Section 5 concludes the article.

## 2. CRKG in Three Typical Duplex Modes

**Figure 3:**
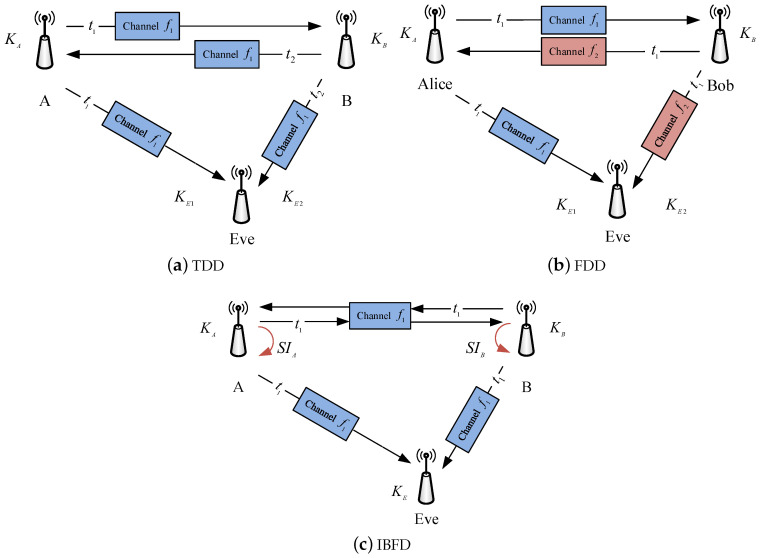
CRKG in three typical duplex modes.

The reciprocity in the propagation of radio waves is the basic premise of CRKG to enable legitimate users to have a common source of randomness, from which they can produce identical secret keys. The duplex mode is essential to the design of CRKG, because channel measurements are carried out by the wireless platforms running in different duplex modes. Figure 3 shows CRKG model in TDD, FDD and IBFD duplex modes. Most CRKG implementations are realized in the TDD mode, in which channel probing is performed alternately. In the FDD mode, Alice and Bob probe the channel at the same time slot, but most of the channel parameters can be quite different due to the frequency separation. For this reason, FDD has received little investigation for CRKG. The drawbacks of half-duplex motivate an exploration of CRKG in IBFD mode, which enable users to probe their channels simultaneously over the same frequency band. Next, we discuss the specific challenges and opportunities of CRKG when it is applied to practical communication systems operating at TDD, FDD and IBFD modes.

### 2.1. Time Division Duplex (TDD) Mode

TDD is wildly used in the existing Long Term Evolution (LTE)-Advanced and emerging 5G New Radio mobile networks, e.g., in TD-LTE, wireless local area networks, Bluetooth and ZigBee. The uplink and downlink transmissions are in different time slots over the same frequency band in TDD systems. The non-simultaneous sampling time is the most challenging problem that hinders the realization of a CRKG scheme in practical TDD systems.

Fortunately, as the uplink and downlink transmission operate at the same carrier frequency, their propagation paths are almost the same if the surrounding environment does not change much between the two channel measurements slots [18,19] and if the transceiver chains for up- and downlink transmission are similar or equal. The channel impulse response (CIR) of a radio wave within the coherence time is considered to be not varying [20,21]. The coherence time is calculated by dividing the coherence length by the phase velocity of light in a medium. For example, the coherence time is about 5 ms when the system runs at a carrier frequency of 2.4 GHz and the moving speed is 10 m/s. If the sampling time difference is deliberately kept smaller than the channel coherence time, the responses of uplink and downlink channels are highly correlated, which satisfies the key generation requirement. Next, interpolation filters are employed to find the value of a signal at unobserved points that lie in between two samples which are known [22]. The reciprocity of interpolated measurements are improved and the effect of the normalized Doppler frequency on the correlation coefficient is reduced. Therefore, many CRKG applications and prototypes have been reported in TDD systems, e.g., by employing WiFi [23,24], ZigBee [25], and Bluetooth [26].

However, it is still a critical open issue to apply CRKG to the TDD scenarios with a very short coherence time. The coherence time is very short in high mobility scenarios, i.e., when terminals move fast or if the multi-path environment changes fast, e.g., moving robots, vehicles, drones, etc. Constrained by bandwidth and switching time, the sampling time difference cannot be infinitely small. When the sampling time difference is significantly greater than the coherence time, the uplink and downlink channel responses have very low similarity. Further investigations are required to address this issue.

### 2.2. Frequency Division Duplex (FDD) Mode

FDD mode is dominant in existing cellular connections, e.g., LTE, NB-IoT, and it is also an indispensable mode in 5G and beyond [27]. In FDD mode, the uplink and downlink transmissions operate at different carrier frequencies simultaneously. Therefore, CRKG in FDD mode is not affected by temporal dynamics and it can support high mobility and high frequency communications. However, the uplink and downlink sub-bands are separated by the frequency spacing, therefore most of the reciprocal channel parameters used in TDD systems, such as received signal strength, channel gains, envelope and phase, can be quite different in FDD systems, depending on the channel coherence bandwidth. It is thus very challenging to find instantaneous reciprocal channel parameters in FDD mode.

One possible solution is to employ frequency-invariant parameters, e.g., the multipath angle and delay [28], which unfortunately can hardly be estimated accurately without massive multiple antennas or wide bandwidth. A second way is to establish combinatorial channels with reciprocal channel gains with the aid of an additional reverse channel training phase, termed as loopback based protocols [29,30,31]. However, these protocols may complicate the channel sounding process and have potential security threats due to passive eavesdropper capturing entire transmissions [32].

Alternatively, we can construct the reciprocal channel parameters by taking advantage of the fact that the propagation paths in the uplink and downlink are reciprocal in most FDD systems [33]. The frequency spacing between the uplink and downlink sub-bands in LTE systems is much less than the center frequency. For example, the center frequency of Band 1 (IMT) is 2100 MHz while the duplex spacing is 190 MHz; the center frequency of Band 30 (WCS) used by AT&T in United States is 2300 MHz while the duplex spacing is only 45 MHz [34]. The duplex spacing is generally less than 10% of the center frequency. Literature and field measurements have shown that the uplink and downlink transmissions travel the same propagation paths and experience the same clusters [27,35]. The frequency spacing leads to the path differences between the uplink and downlink, especially to the phases. The observed channel information would be different due to the superposition of multipath effects. Different channel paths should thus be separated by wideband or massive antennas in order to extract the reciprocal channel parameters. According to the uplink and downlink frequencies, some compensation methods are used to remove or decrease the differences, and extract the reciprocal parameters to generate the secret key. Further studies are still required to model and prototype CRKG schemes, when the frequency spacing is large.

### 2.3. In Band Full Duplex (IBFD) Mode

IBFD communications enable users to transmit and receive simultaneously over the same frequency band. Therefore, it is not constricted by aforementioned problems in TDD and FDD modes. Even without considering these non-reciprocity issues, it also has attracted significant interest to study CRKG in IBFD mode, because it offers the potential to double the key rate, as measured by the number of secret key bits generated per second per Hz. In some scenarios, the growth may be slightly lower than double, due to the time correlation of adjacent channel measurements. Furthermore, IBFD provides additional protection against eavesdroppers because they observe the superposition of simultaneous transmissions over the two-way channel.

Due to the close proximity of the transmitter and the receiver antennas, simultaneous transmission and reception of signals emanates a key issue of self-interference (SI) [36]. For a full-duplex base-station to achieve the link SNR equal to that of a half-duplex counterpart, it needs to suppress SI by about 106 dB. Therefore, some residuals will remain in practice. The inevitable residual self-interference (RSI) is asymmetric due to different self-interference channels and the hardware deviation. Therefore, it is the most challenging issue that hinders the practical implementation of CRKG in IBFD mode. One solution comes from the fact that the SI channel changes slower than the channel between users. At this point, theoretical CRKG approaches in IBFD mode are proposed in [37,38], while practical CRKG testbed with IBFD capability universal software radio peripheral (USRP) devices and near field communication (NFC) devices are demonstrated in [38,39], respectively. IBFD communications can noticeably boost the secret key rate in an operationally interesting regime of parameters.

**Figure 4:**
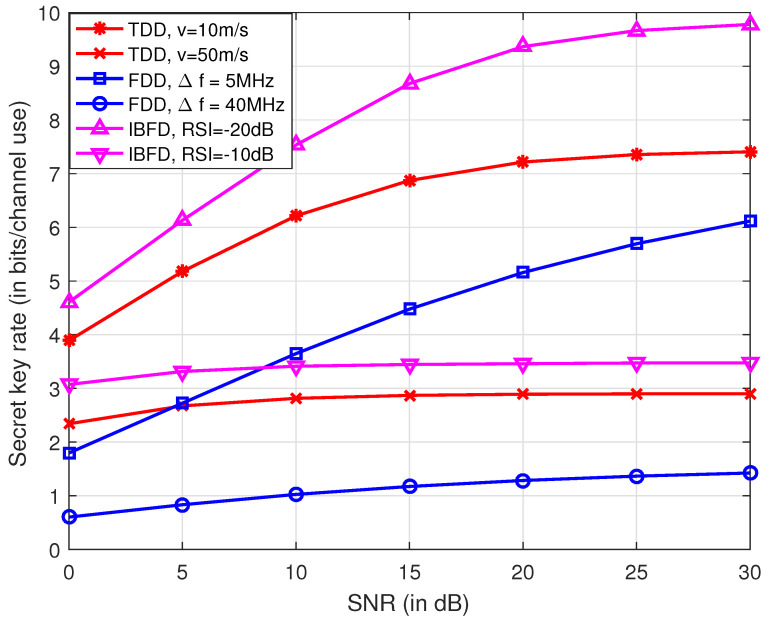
Comparison of secret key rates for different duplex modes. In TDD systems, the time interval between the uplink and downlink is 0.5 ms with velocities of 10 m/s and 50 m/s. In FDD system, the center carrier frequency is 2 GHz, and the frequency interval is 5 MHz and 40 MHz. Under IBFD operation, the power of RSI is set −10 dB and −20 dB less than that of the received signals.

Figure 4 shows the secret key rate for systems operating at TDD, FDD and IBFD modes. It is observed in the figure that, with lower mobility, smaller frequency separation and smaller RSI, TDD, FDD and IBFD achieve higher secret key rate, respectively. It is also seen that with appropriate techniques for SI cancellation, that IBFD outperforms its half duplex counterparts. The secret key rate is calculated as bits per channel use since the channel impulse response within the coherence time is considered to be not varying. Mobility poses a negative effect on channel reciprocity for TDD mode, and thus the secret key rate for *v* = 50 m/s is lower than that of *v* = 10 m/s. However, the channel sampling rate can be improved for high mobility scenarios, since the coherence time is reduced. The secret key rate per unit time might increase, which will be studied in the future.

## 3. CRKG in Massive MIMO and mmWave Systems

Several key enabling technologies have been identified, such as massive MIMO and mmWave communications, to achieve high throughput and serve a large number of terminals in the 5G and beyond wireless networks. In massive MIMO systems, one base station (BS) employs a large number of (e.g., hundreds of) antennas simultaneously serving several (e.g., tens of) user terminals in the same time frequency resource. By employing a large number of antennas, energy can be focused into small spatial regions, providing high spatial resolution and reducing intra-cell interference. Hence, massive MIMO can significantly improve the spectral efficiency and power efficiency. The performance bottleneck is the pilot contamination caused by the pilot reuse [40].

MmWave communications is another key technology to meet the explosive user data demand for the future wireless communications. It corresponds to 30–300 GHz frequency bands to support data rates of multiple gigabits per second. Compared with sub 6 GHz bands, mmWave bands provide large available bandwidths. Moreover, thanks to small wavelength at mmWave frequencies, a large number of antennas can be packed into a physically limited space. For example, the number of antennas at 70 GHz can go up to 1024 at the base station and 64 at the user equipment [41]. In addition, without spatial coding gain, the attenuation at mmWave is too large. Thereby, mmWave communications are usually combined with massive MIMO technology.

In massive MIMO and mmWave communication systems, the channel exhibits some novel characteristics, bringing opportunities and challenges to CRKG and opening a new and promising area.

### 3.1. Massive MIMO

The large scale antenna array in massive MIMO systems offers high spatial dimension for the key generation. As the number of antennas increases, the signals are precoded to be concentrated toward one or more directions [42]. The narrow and directional beam increases the received SNR, as well as the secret key rate. Moreover, the narrow beam weakens the signal reception at the passive eavesdropper significantly.

On the other hand, CSI acquisition is a challenging problem in massive MIMO systems. Orthogonal pilots are usually used to estimate the CSI and their length scales with the number of transmit antennas. When the number of antennas at the BS is large, it is impractical to estimate the downlink CSI at the user terminal side. Besides, eavesdroppers may utilize the pilot contamination attack [43], where the eavesdropper transmits the same pilot signals as the legitimate user. Then, the BS obtains the summation of the channel legitimate user and that of the eavesdropper, impacting the reciprocity of the channel observations of the BS and user terminals.

Fortunately, the spatial sparse property of massive MIMO channels provides a potential solution [42,44,45]. With a massive antenna array, the BS can distinguish propagation paths from different directions. By preprocessing, the channel matrix (or vector) reveals the sparse property, where a few dominant elements represent the channel gains of different paths and contain the most channel energy [44,45]. Then, the BS and user terminals can estimate their effective channel matrices with low dimensions, containing the dominant channel path information. For the pilot contamination attack, there exists a crucial complementary relation between the received signal strengths at the eavesdropper and the legitimate user [43]. This relation can measure the amount of information leakage to the eavesdropper and a rate-adaptation scheme is proposed.

**Figure 5:**
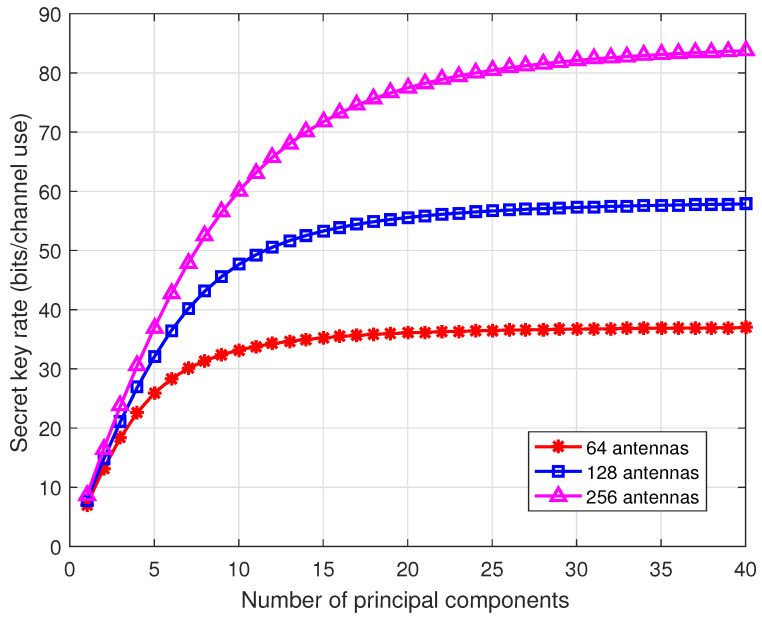
Secret key rates for different number of antennas. Consider the TDD communications systems with suburban macro scenario, where the center carrier frequency is 2 GHz, and the velocity is 1 m/s. The SNR is 10 dB.

Figure 5 illustrates the secret key rates as the number of antennas increases. Consider 64, 128, and 256 antennas at the BS and single antenna users. When the antenna array at the BS is uniform linear array (ULA), we can employ the discrete Fourier transform (DFT) matrix to transform the channel vector into the beam domain [45]. In the beam domain, principle components or dominant beams of the channel vector contain most of the channel power. We calculate the secret key rate of the 40 strongest principal components. From the results, by extracting a few (e.g., 10–20) components, the secret key rate well approaches that of the whole channel. Thus, in the massive MIMO communications, we only need to estimate the principal components of the beam domain channel vector, significantly reducing the pilot overhead.

### 3.2. MmWave Communications

MmWave communications are usually combined with a large number of antennas. Specially, the BS and the user sides are both equipped with massive antennas. Thus, the spatial sparse property of mmWave massive MIMO channel still holds in [46]. Different from the conventional sub 6 GHz wireless channels, the mmWave massive MIMO channels have some different propagation characteristics. The main novel channel properties include the non-stationary property over antenna array axis [47]. Some measurement results has shown that the multipath components parameters may vary over the large physical array, e.g., received power, delay spread, and azimuth angular spread [47]. The new channel characteristics provide more channel temporal-spatial parameters to generate the secret keys.

The mmWave communications also bring new challenges due to the path and penetration loss [48]. Along with the frequency growth, the mmWave channel suffers severe path loss. The path loss in free space is proportional to the square of the frequency. Because the frequency of mmWave signals is much higher than conventional microwave communications, it leads to higher path loss. Penetration loss is another propagation loss. While sub 6 GHz signals penetrate through some objects, mmWave signals are easily blocked by most solid materials. Even for tinted glass, the penetration loss is high for 28 GHz signals. These two propagation losses lead to lower numbers of propagation paths, and thereby independent stochastic sources. Specifically, when the propagation experiences a significant line of sight, the channel randomness is limited.

The directional beamforming provides a potential solution to the path loss challenge. The narrow beams concentrate the transmitted power to the directions of the user, compensating the path loss and improving the received SNR. For low number of channel paths, the work in [49] chooses randomly generated perturbations to increase the secret key rate. After the initialization of the communication link, the BS and user know the transmitted and received angles. Then, a transmitter defined randomness (a perturbation angle) is integrated into key generation. Moreover, the non-stationary channel property also allows generating secret keys from the spatial randomness.

## 4. Bringing CRKG into the Field: Securing Practical IoT Communications

Secure data exchange among devices and users is the prerequisite for the successful development and deployment of IoT infrastructures in industrial environments. Holistic security concepts are required to prevent vulnerabilities, enable remote access and control the production systems. On the one hand, due to low cost requirements of sensor nodes and the massive number of IoT devices, the conventional key distribution schemes have been challenged. On the other hand, CRKG has been prototyped to establish cryptographic keys and safeguard IoT connections. This section reviews these prototypes and analyzes the feasibility of CRKG for heterogeneous IoT devices interconnected with various short-range and long-range communication solutions. These heterogeneous wireless devices follow the same standards, but may interpret received signal strength indication (RSSI) in different ways [50]. However, because each key generation party carries out the quantization independently, the key generation process can still be feasible with heterogeneity of devices [51]. Finally, we discuss the remaining challenges and research directions in the domain.

### 4.1. Short-Range IoT Communication Solutions

#### 4.1.1. IEEE 802.15.4/Zigbee

The IEEE 802.15.4 standard defines the physical and medium access control (MAC) layer for low-data rate and low-power transmissions, and ZigBee is a full communication suite based on the IEEE 802.15.4.

**Figure 6:**
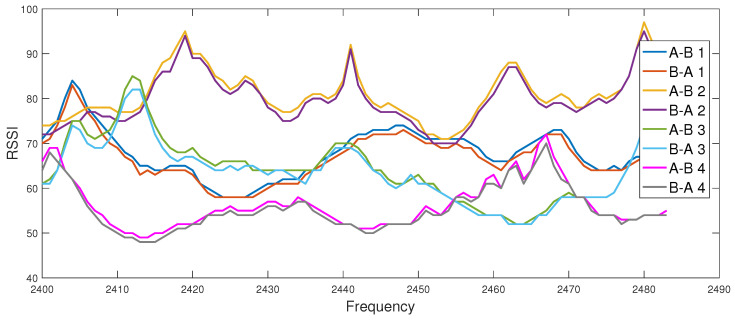
Example channel measurements of IEEE 802.15.4 transceivers between 2.4 and 2.483 GHz in office environment: RSSI over frequency of three reciprocal links.

Within the fast-secure project (de.fast-zwanzig20.de/industrie/fast-secure/), standard-conform USB dongles from metraTec, which realize the 802.15.4 standard, are applied. The hardware supports the ISM-band between 2.4 and 2.483 GHz. Periodically with 1 MHz, a beacon is sent by the devices to check connectivity. One measurement takes about 12 ms, while one complete sweep over all 83 MHz takes about 1.5 s. Based on a remote signal, the devices perform a measurement of the channel reported on the serial interface to the host laptop. Figure 6 shows a good agreement between the channel measurements of Alice and Bob in both directions for four trials (A-B represents the channel from Alice to Bob and B-A represents the channel from Bob to Alice). The applicability of CRKG is also investigated for the field of automation systems using channel parameters recorded in a real industrial environment [25]. Up to now, low secret key rates are reported to support low data rate links.

#### 4.1.2. Ultra-Wideband

**Figure 7:**
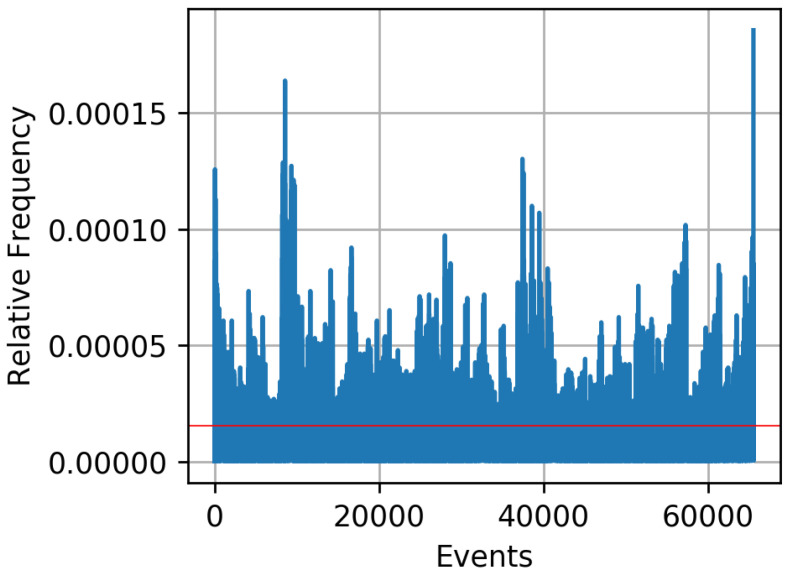
Relative frequency of the single events with different processing approaches at 16 bit with preprocessing according to [52] and RBA. The red line shows the “perfectly” uniform distribution achieving maximum entropy.

Ultra-wideband technique uses a very low energy to achieve short-range, high-bandwidth communications. In [52], two wireless sensor nodes measure the reciprocal CIR using ultra-wideband signals with 500 MHz bandwidth in the 4 GHz band. Messages are transmitted with impulse radio ultra-wideband, which supports short pulses in the time domain, with a typical duration of less than 3 ns. The received preamble sequence is correlated against the expected preamble sequence to estimate the CIR. Alice and Bob transmit messages under line-of-sight conditions in a laboratory and in the adjacent hallway. After reception of a packet, they estimate their CIR. Alice and Bob are stationary and 5 m apart. The receivers are placed 1 m above ground to ensure optimal transmission quality. The line-of-sight path is interrupted when people move along causing random changes of the CIR. Alice and Bob transmit messages every 370 ms, generating six CIRs per second. One single measurement consists of 200 data points. Measurements were performed for six days, generating more than 20 GB of data. Depending on the signal processing for bit extraction and key agreement, secret key rates in the order of up to 12 bits per channel use are reported in [52]. Using Results-Based-Adaption (RBA) in order to adapt the quantization decision so that its outcome is as close as possible to the uniform distribution, reasonable relative frequencies of the resulting 16-bit sequences is illustrated in Figure 7.

#### 4.1.3. IEEE 802.11/WiFi

IEEE 802.11/WiFi is the dominant wireless technique for the local area networks, which is incorporated in almost all smartphones, laptops, tablets, etc. IEEE 802.11 defines the physical layer and MAC layer primitives, and many of these features are beneficial for key generation.

IEEE 802.11 adopts different physical layer modulation techniques, including OFDM, DSSS, and MIMO. For all the physical layer modulations, RSSI at the receiver represents the channel quality and is available at all commercial WiFi transceivers, which makes it the most popular channel parameter for CRKG. In addition, OFDM is used in the IEEE 802.11a/g/n/ac, which provides high data rate with high spectral efficiency. The frequency domain channel estimation can be obtained by transmitting training symbols. This results in finer-grained channel information and allows capturing more channel randomness. However, channel estimation is not made public in many transceivers and specialized software defined radio platforms are required for access. The prototypes of IEEE 802.11 have been developed under both TDD and IBFD mode.

Distributed coordination function (DCF) is the key component of the IEEE 802.11 MAC protocol. The receiver sends an acknowledgment (ACK) packet for each received data packet to confirm the successful reception. The time interval between these two transmissions is very low, in the order of microseconds. On the other hand, WiFi usually operates in the indoor environments when the channel experiences slow fading. The channel variation is usually introduced by the movement of humans, e.g., with speeds up to two meters per second. Therefore, the data and ACK packets can be leveraged to obtain highly correlated channel measurements. It is worth noting that key generation does not require extra and dedicated transmissions but can be achieved based on existing packets, therefore, the overhead can be kept at minimum.

**Figure 8:**
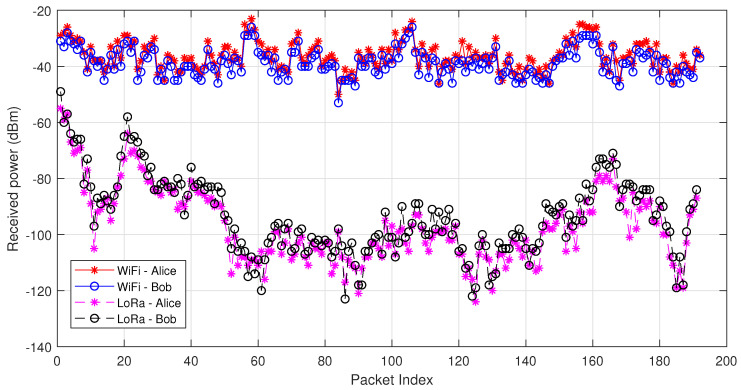
Received power of Alice and Bob operating with WiFi and LoRa.

Some experimental results are shown in Figure 8, with IEEE 802.11g (OFDM) tested. The testbed consists of two WARP boards [53] and the experiments are carried out in an indoor office environment. The received power of Alice and Bob is shown as an example, where high correlation between the channel measurements of Alice and Bob can be observed.

### 4.2. Long-Range IoT Communication Solutions

#### LoRa

LoRa is a new IoT technique and has been used widely in smart agriculture, smart cities, etc. It uses chirp spread spectrum (CSS) to modulate the signals, which is immune to multipath and Doppler shift, and has a very broad receive sensitivity, as low as −148 dBm. Therefore, LoRa can support long range communications, namely up to 15 km when the channel quality is good.

Similar to many other wireless techniques, LoRa is also working in the TDD mode, which is ideal for CRKG. The receiver calculates the power for each received transmission, through which the channel randomness can be exploited. LoRa extends key generation applications to unexplored long range communication scenarios [54,55,56,57]. For example, the distance between Alice and Bob is as high as 1.5 km in the experiments of [56].

A LoRa-based key generation system is designed in [54]. The LoRa node consists of an Arduino and a LoRa Shield which uses SX1276 as the LoRa transceiver. RadioHead [58], an Arduino-based LoRa library is used. A typical LoRa setup is considered, where one LoRa device remains stationary, emulating the gateway, while the other one moves around in an urban environment with a maximum communication range as 500 m. As shown in Figure 8, the received power varies from −50 dBm to −122 dBm. Compared to the results of WiFi-based key generation, LoRa-based key generation has a much larger variation of received power. The conventional absolute value-based quantizer, e.g., mean-based quantization, generates long series of 1s and 0s; however, an adaptive version requires learning the channel variation in real time, which results in additional overhead. A differential-based quantizer is used in [54], but each comparison only leads to one key bit.

### 4.3. Open Research Challenges

- **Narrow-band IoT (NB-IoT).** NB-IoT is a new technology standard, designed to broaden the future of IoT connectivity. However, no CRKG prototype has been developed for NB-IoT yet. One major issue is that Release 13 NB-IoT only supports FDD mode.
- **Protocol limitation.** Some IoT protocols are not flexible to provide ideal condition for channel sounding. For example, LoRaWAN is the most popular MAC layer protocol for LoRa and the class-A is compulsory for all the implementation. There will be a one-second interval between the uplink and downlink transmissions, which will impact the correlation of the channel measurements.
- **Static Devices.** Many IoT devices, e.g., unattended devices, can be fixed and the channel randomness is limited in this case. The channel variations induced by the environment changes might not be significant and it is difficult for the low cost IoT devices to be sensed. This challenge can be tackled by leveraging beamforming, helper devices or artificial randomness [59].
- **Frequency Hopping.** Frequency hopping spread spectrum (FHSS) technology is widely used in military and civilian IoT communications, e.g., Bluetooth. However, it is very challenge to apply CRKG in a FHSS system because the devices change the carrier frequency and the channel reciprocity is impacted.
- **Massive Connectivity.** The performance of CRKG is impaired for massive connectivity scenarios. The overhead of CSI acquisition scales with the number of IoT devices and becomes extremely large with massive IoT devices. Meanwhile, some IoT applications only transmit short packages. The huge overhead makes CRKG impractical. Besides, when non-orthogonal pilot signals are employed among different devices, it may bring interference to degrade the correlation of channel observations.

## 5. Conclusions

This paper identifies opportunities and discusses technical challenges in the channel reciprocity-based secret key generation driven by different duplex modes, massive MIMO and mmWave communications, and prototypes in IoT networks. These are promising technologies in the 5G and beyond wireless networks supporting ultra high data rate and massive number of devices. Moreover, we discuss the potential solutions to cope with these challenges. The extension of physical layer secret key generation into new areas of future wireless networks will further stimulate physical layer-based security schemes and techniques.

## Figures and Tables

**Table 1:** Brief summaries on existing surveys/tutorials.

Surveys/Tutorials	Publications	Focused Issues
Ren et al. [8]	IEEE Wireless Communications	Fundamental and general framework of CRKG, protocols and performance comparison of received signal strength-based and channel phase-based CRKG.
Zeng et al. [9]	IEEE Communication Magazine	Security issues of CRKG under practical passive and active attacks and countermeasures.
Zhang et al. [10]	IEEE Access	Comprehensive overview on the principles, performance metrics, procedures and applications of CRKG.
Zhang et al. [6]	Entropy	Physical layer key generation and physical layer encryption in the IoT.

## Abbreviations

The following abbreviations are used in this manuscript:

5G	The fifth generation
ACK	Acknowledgment
BS	Base station
CIR	Channel impulse response
CRKG	Channel reciprocity-based key generation
CSI	Channel state information
CSS	Chirp spread spectrum
DCF	Distributed coordination function
DFT	Discrete Fourier transform
FDD	Frequency division duplex
FHSS	Frequency hopping spread spectrum
IBFD	In-band full-duplex
IoT	Internet of things
LTE	Long term evolution
MAC	Medium access control
MIMO	Multiple-input multiple-output
NB-IoT	Narrow-band IoT
NFC	Near field communication
RBA	Results-based-adaption
RSI	Residual self-interference
RSSI	Received signal strength indication
SI	Self-interference
TDD	Time division duplex
ULA	Uniform linear array
USRP	Universal software radio peripheral
